# Functional crosstalk in culture between macrophages and trigeminal sensory neurons of a mouse genetic model of migraine

**DOI:** 10.1186/1471-2202-13-143

**Published:** 2012-11-21

**Authors:** Alessia Franceschini, Asha Nair, Tanja Bele, Arn MJM van den Maagdenberg, Andrea Nistri, Elsa Fabbretti

**Affiliations:** 1 Department of Neuroscience and Italian Institute of Technology Unit, International School for Advanced Studies (SISSA), Via Bonomea 265, Trieste, 34136, Italy; 2 Departments of Human Genetics & Neurology, Leiden University Medical Centre, Leiden, RC, 2300, The Netherlands; 3 Center for Biomedical Sciences and Engineering, University of Nova Gorica, Nova Gorica, SI-5000, Slovenia; 4 Present address: Department of Experimental and Diagnostic Medicine, Section of General Pathology, University of Ferrara, Ferrara, Italy

**Keywords:** P2X3 receptor, Purinergic receptor, Pain, Neuroinflammation, ATP, Sensitization

## Abstract

**Background:**

Enhanced activity of trigeminal ganglion neurons is thought to underlie neuronal sensitization facilitating the onset of chronic pain attacks, including migraine. Recurrent headache attacks might establish a chronic neuroinflammatory ganglion profile contributing to the hypersensitive phenotype. Since it is difficult to study this process *in vivo*, we investigated functional crosstalk between macrophages and sensory neurons in primary cultures from trigeminal sensory ganglia of wild-type (WT) or knock-in (KI) mice expressing the *Cacna1a* gene mutation (R192Q) found in familial hemiplegic migraine-type 1. After studying the number and morphology of resident macrophages in culture, the consequences of adding host macrophages on macrophage phagocytosis and membrane currents mediated by pain-transducing P2X3 receptors on sensory neurons were examined.

**Results:**

KI ganglion cultures constitutively contained a larger number of active macrophages, although no difference in P2X3 receptor expression was found. Co-culturing WT or KI ganglia with host macrophages (active as much as resident cells) strongly stimulated single cell phagocytosis. The same protocol had no effect on P2X3 receptor expression in WT or KI co-cultures, but it largely enhanced WT neuron currents that grew to the high amplitude constitutively seen for KI neurons. No further potentiation of KI neuronal currents was observed.

**Conclusions:**

Trigeminal ganglion cultures from a genetic mouse model of migraine showed basal macrophage activation together with enhanced neuronal currents mediated by P2X3 receptors. This phenotype could be replicated in WT cultures by adding host macrophages, indicating an important functional crosstalk between macrophages and sensory neurons.

## Background

Migraine is a neurological disease whose pathophysiological mechanisms are still incompletely understood. Genetic research into rare monogenic forms of migraine such as familial hemiplegic migraine (FHM) has advanced our knowledge hinting at increased neuronal excitability as a major factor in disease etiology [1] that may be applicable to common types of migraine as well. Little is, however, known about the factors sensitizing patients to pain. It has been hypothesized that long-term alterations in trigeminal ganglion function may predispose a patient to subsequent attacks [2]. Previous studies have produced a genetic mouse model that carries a missense R192Q mutation in the α1 subunit of neuronal Ca_V_2.1 (P/Q-type) Ca^2+^ channels that causes familial hemiplegic migraine type 1 (FHM-1) in patients [3,4]. Knock-in (KI) mice carrying this mutation show channel gain-of-function with larger neuronal calcium influx, facilitated neurotransmitter release and lowered threshold for cortical spreading depression [4-7], a wave of neuronal and glial depolarization that underlies the migraine aura [8]. Furthermore, it has been shown that trigeminal ganglion nociceptive neurons of R192Q KI mice possess enhanced responses mediated by P2X3 receptors [9] that are thought to be important neuronal transducers of pain signals [10].

In mouse trigeminal ganglia in situ, the R192Q mutation causes also changes in non-neuronal cells like satellite glial cells [11]. These experimental observations are in line with the theory of sterile inflammation that predicts, at meningeal level, local release of substances activating nociceptive sensory neurons as a possible mechanism for headache attack in migraine [12,13]. Accordingly, trigeminal sensory fibers (that innervate the dura mater and transmit nociceptive signals to the brain) would operate in an inflammatory milieu characterized by increased vascular permeability, vasodilation, and local oedema [12,13]. This process may establish a repeating cycle of headache attacks [14]. Although this theory has been criticized [15], there are no data firmly excluding neuroinflammation as a process to sensitize trigeminal sensory neurons.

Macrophages are, in general, major players in inflammatory conditions as their responses to changes in the microenvironment can determine disease outcome [16], and may even represent a therapeutic target to reverse or prevent pathological reactions. Thus, the present study explored the possibility that macrophages may have a role in tissue sensitization like the one observed in certain chronic diseases [12]. In particular, the study aim was to test any crosstalk between macrophages and P2X3 receptor-expressing neurons (comprising the largest neuronal majority in the ganglion and in culture; [17]) that might induce functional changes in single macrophage activity or in single neuron currents. To this end, using trigeminal ganglion cultures, experiments were performed by adding known numbers of host macrophages and examining the morphological and functional consequences.

## Methods

### Animals

Ca_V_2.1 R192Q knock-in (KI) and wild-type (WT) mouse littermates were used [4]. All experimental procedures were in strict accordance with the Italian and EU regulation on animal welfare and were previously approved by the SISSA ethics committee. Mouse genotyping was performed as previously reported [4].

### Culture procedure and protocol for macrophage-trigeminal ganglion co-cultures

Trigeminal ganglion cultures were obtained from P12-14 mice as described before [17] and, after 48 h from plating, employed for patch clamping, macrophage functional tests or molecular/cell biology experiments. From each mouse two ganglia were excised and used to prepare one petri dish. Control and test cultures were always run in parallel on the same day.

To explore the potential interaction between macrophages and trigeminal ganglion cells, in a series of experiments, we supplemented standard trigeminal ganglion cultures with the addition of host macrophages. To this end, mouse host macrophages (MФ) were extracted from the peritoneal cavity of WT adult mice 72 h after a single intraperitoneal injection of Brewer thioglycollate medium (0.4 g/kg, 3% wt/vol, Sigma, Milan, Italy) [18]. Primary macrophages were plated in DMEM/10% FBS medium and kept for 14 d in culture (with change of medium every 48 h). Thereafter, macrophages were collected, counted in a Burker chamber, and transferred (as a batch of 120,000 cells), for co-cultures experiments, to Petri dishes containing trigeminal ganglia (from WT or R192Q KI mice) dissociated on the same day. Experiments were performed after 2 d of co-culturing. For the ATP assay and Fluoresbrite® YG latex Microspheres experiments, we used multiwall plates in which an equivalent number of macrophages (30,000 cells/cm^2^) were plated together with trigeminal neurons (from WT or R192Q KI mice) for co-cultures experiments. We estimated that this addition increased the percent of macrophages from about 3–5 to 10–15% of the total DAPI-positive cell population in a region of interest (ROI) of 640 × 480 μm. It should be noted that, under the present experimental conditions, it was not possible to distinguish host macrophages from resident ganglion macrophages.

### Immunofluorescence microscopy

Immunocytochemistry of trigeminal ganglia in culture from WT or R192Q KI mice was performed as already described [17]. For immunocytochemistry of trigeminal ganglia in situ, a series of 14 μm-thick longitudinal sections of trigeminal ganglia was collected. The following antibodies were used: anti-β-tubulin III (1:1000; Sigma) for neuronal staining, and anti-Iba1 (1:300, Wako, Osaka, Japan), a microglia/macrophage-specific marker of macrophage lineage in central and peripheral neuronal tissue [19,20]. For secondary immunostaining AlexaFluor goat-anti-mouse 488- or goat-anti-rabbit 594-conjugated antibodies (1:300 Invitrogen; S.Giuliano Milanese, Italy) were used; nuclei were counterstained with DAPI (1:1000, Sigma). Images in a 640 × 480 μm ROI were visualized with a Zeiss Axioskop fluorescence microscope (Zeiss, Zurich, Switzerland), and analyzed with either MetaMorph software (Molecular Devices, Downingtown, PA, USA) or ImageJ software (NIH, USA) with ITCN plugin. 3D reconstructions (Z-stack; 0.5 μm steps) of high magnification confocal images (Leica TCS SP2, Wetzlar, Germany) of intact ganglia were obtained with ImageJ software and quantified with ImageJ Voxel counter (voxel, μm^3^).

### Phagocytosis assay

Macrophage phagocytosis tests were performed by incubating cultures with FITC-conjugated Zymosan A (Zy-FITC; 1 mg/ml, Sigma) for 10 min at 37°C, fixed in 4% paraformaldehyde and processed for immunofluorescence [21]. Active macrophages were considered when taking up ≥ 1 granule of Zy-FITC. After counting the average number of granules/active macrophage, the phagocytosis index was calculated as the percentage of Zy-FITC-positive macrophages multiplied by the number of Zy-FITC granules per single cell [22]. Experiments were also performed with Fluoresbrite® YG latex Microspheres (1.00 μm, PolySciences, Warrington, PA, USA) on trigeminal neuronal cultures and macrophages co-cultures (incubation was 15 min at 37°C). Fluorescence signals were detected with a Perkin Elmer fluorimeter at 488 nm wavelength. Cumulative probability plots were constructed as shown before [23].

### Real time - PCR and protein analysis

Total mRNA was extracted from cultures of WT or KI mouse peritoneal macrophages as described before [8]. Real-time PCR reactions were run in duplicate in an iQ5 thermocycler using IQ SyBr Green Supermix Reactions (Bio-Rad Hercules, CA, USA), with specific primers for P2X3 [9] or Cav2.1 (Fw: 5^′^-GAAGTCCATCATAAGTCTGTTGTT-3^′^ and Rw: 5^′^- GCCACCGAACAGCTGCAT-3^′^) [24]. All primer sequences were designed using Beacon designer (PREMIER Biosoft International, Palo Alto, CA, USA) and were previously validated [8,17]. Calculations for relative mRNA transcript levels were performed using the comparative method between cycle thresholds of different reactions [8,17]. Quantitative PCR was performed in duplicate following the MIQE guidelines [25].

Western blotting was performed as described earlier [8,16], using antibodies against anti-P2X3 (1:300; Alomone, Jerusalem, Israel), anti-β-tubulin III (1:2.000; Sigma) or anti-actin (1:3.000; Sigma). Grey values were quantified with Scion Image software (Scion, Frederick, Maryland, USA) or Uviband (Uvitec, Cambridge, UK). Total protein content of ganglia was measured with the BCA kit purchased from Sigma.

### ATP release assay

Basal ATP concentrations in the extracellular medium collected from 24 h trigeminal ganglion (WT or KI) cultures were measured with ENLITEN ATP Assay (Promega, Italy), according to the manufacturer’s instructions. Extracellular ATP was also measured in the medium of macrophage-neuronal co-cultures following the addition of 30,000 host macrophages /cm^2^ (5 h).

### Patch-clamp recording

P2X3 receptor-mediated currents were recorded, under patch clamping conditions in whole-cell configuration, from trigeminal neurons obtained from WT or R192Q KI mice, using the specific agonist α,β-methylene-ATP (α,β-meATP) applied with a fast superfusion system (Rapid Solution Changer RSC-200; BioLogic Science Instruments, Claix, France). Full details of the electrophysiological methods have been previously reported [8,17]. Trigeminal neurons were superfused continuously (2 mL/min) with physiological solution containing (in mM): 152 NaCl, 5 KCl, 1 MgCl_2_, 2 CaCl_2_, 10 glucose, and 10 HEPES (pH adjusted to 7.4 with NaOH). Patch pipettes (3–5 MΩ resistance) were filled with the following solution (in mM): 140 KCl, 0.5 CaCl_2_, 2 MgCl_2_, 2 Mg_2_ATP_3_, 2 GTP, 10 HEPES, and 10 EGTA (pH adjusted to 7.2 with KOH). Cells were held at −60 mV. Data were filtered at 1 KHz and acquired by means of a DigiData 132XInterface and pClamp 8.2 software (Molecular Devices, Sunnyvale, CA, USA). We measured current peak amplitude, current rise-time (10–90% of peak amplitude), onset of desensitization (estimated by calculating the first-time constant of current decay, τ_fast_), and recovery from desensitization (with paired-pulse experiments in which α,β-meATP applications were spaced at 30-s interval). Recovery was expressed as % of the first response in each pair [9,17].

To find out how WT P2X3 receptor currents might have been affected by the number of host macrophages, we compared current amplitudes after adding 120,000 or 300,000 or 1 million MФ for 48 h to the primary ganglion cultures. Under these conditions, while control WT currents had an average amplitude of −290 ± 30 pA (*n* = 15), co-culturing with 120,000 host macrophages raised the average amplitude of P2X_3_ currents to −500 ± 60 pA (*n* = 24; *p* < 0.05). Larger macrophage numbers (300,000 or 1 million) led to current amplitudes of −660 ± 90 (*n* = 6) or −540 ± 70 pA (*n* = 7), values that were not significantly different from the one observed with 120,000 macrophages. Hence, further experimental tests were routinely carried out with 120,000 host macrophages.

### Statistics

Data are expressed as mean ± standard error of the mean (SEM), where *n* indicates the number of independent experiments or the number of investigated cells. Statistical analysis was performed using the Student’s *t*-test, or the Mann–Whitney rank sum test after the software-directed choice of parametric or non-parametric data, respectively (Sigma Stat and Sigma Plot, Systat Software Inc., San Jose, CA, USA). A *p* value of ≤ 0.05 was accepted as indicative of a statistically significant difference.

## Results

### Strong phagocytotic activity of KI macrophages *in vitro*

**Figure 1:**
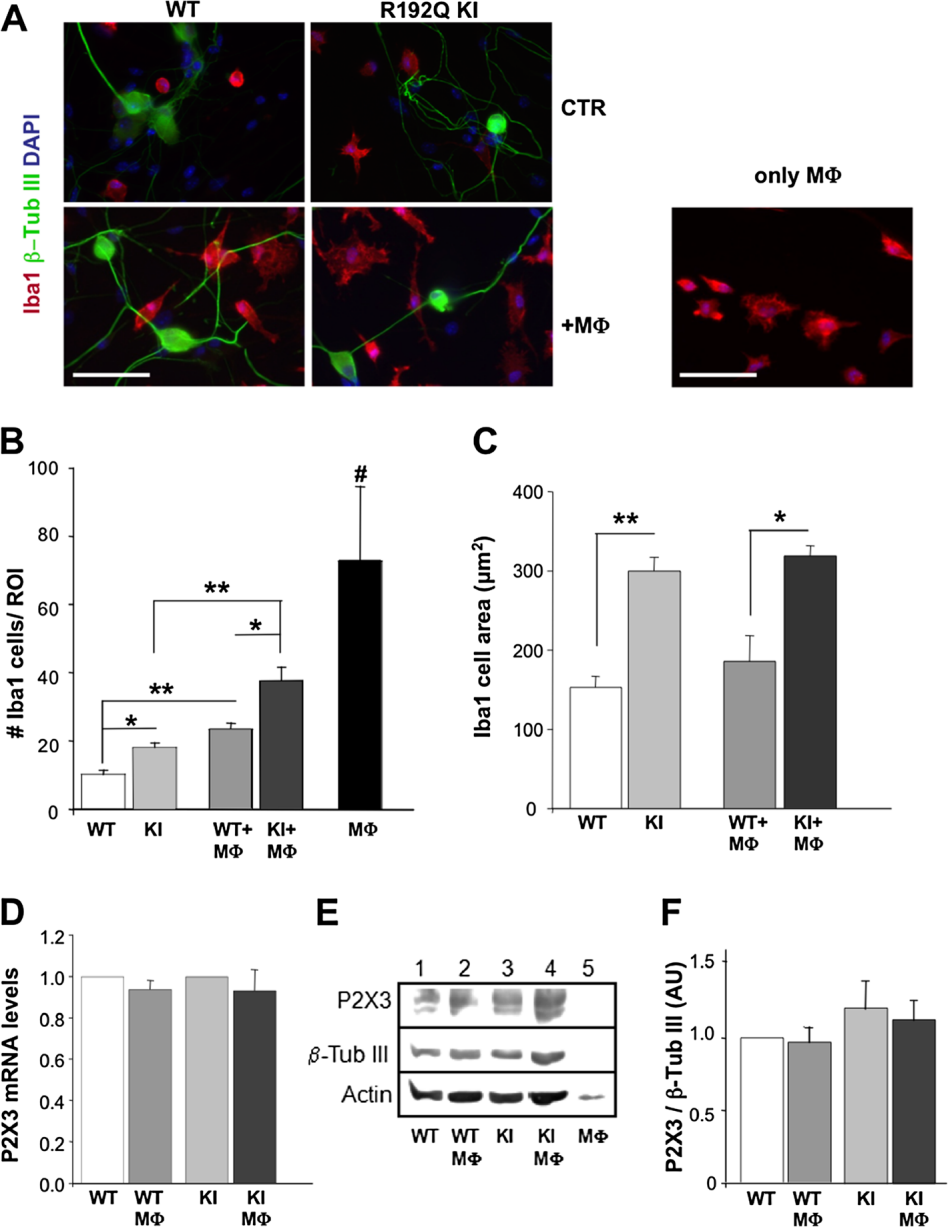
**Activated phenotype of trigeminal ganglion macrophages *in vitro. A***, Examples of fluorescence microscopy images of cultures from WT or KI trigeminal ganglia immunostained with antibodies against Iba1 (red), β˜-tubulin III (green) and counterstained with DAPI (blue). MФ. indicates co-culturing with known number of host macrophages, while only MФ .indicates pure peritoneal macrophage cultures. Scale bar: 50 μm. ***B***, Histograms quantify the number of Iba1-positive cells in the ROI (640 × 480 μm) from WT or R129Q KI ganglion cultures in control condition and after MФ. addition; *n* = 3 petri dishes for each group run in parallel, * *p* < 0.05, ** *p* < 0.01. MФ .# *p* < 0.01 vs all conditions. ***C***, Histograms quantify the average cell area (expressed in μm^2^) for data shown in ***B***, * *p* < 0.05, ** *p* < 0.01. ***D***, P2X3 mRNA levels in co-cultures (WT+MФ, KI+MФ) expressed as fold increase in comparison to their controls (WT or R192Q KI cultures) and normalized versus β-tubulin mRNA levels (n = 3 petri dishes for each group). ***E***, Example of western blots shows similar levels of P2X3 receptor expression in lysates from WT or R192Q KI trigeminal cultures (lanes 1 and 3), and in co-cultures (WT+MФ and KI+MФ, lanes 2 and 4). Lysates from MФ only (lane 5) show no signal. β-Tubulin III and actin are shown as loading control (bottom row). ***F***, Histograms show mean values (optical density, AUs) of P2X3 subunits obtained in western blot experiments normalized over β-tubulin III signals; *n* = 4 petri dishes.

Figure 1A compares representative images of trigeminal ganglion cultures (from WT or R192Q KI mice) in which β-tubulin III-positive neurons (green) and Iba1-positive macrophages (red) are shown. In basal conditions, macrophages were a rather small component of the cell population in culture since they were 3.9 ± 0.3% (*n* = 7 petri dishes) of DAPI positive elements in WT cultures, and 5.2 ± 0.4% (*n* = 7; *p* < 0.05) in R192Q KI ones. The absolute number of macrophages in the ROIs was significantly higher in R192Q KI cultures in comparison to WT ones (Figure 1A,B; * *p* < 0.05). R192Q KI Iba1-positive cells also possessed larger area (Figure 1C) that is a standard index of macrophage activation state [26]. Thus, the present results indicated that KI cultures after 48 h contained a larger number of macrophages probably in their activation state. 

Similar observations were obtained from sections of acutely-excised trigeminal ganglia. Thus, Iba1 immunoreactivity was 1.6 ± 0.1% /area for KI trigeminal ganglion sections vs 0.70 ± 0.05% /area for WT sections (n = 3; p < 0.001). It is important to note that satellite glial cells are Iba1 negative in trigeminal ganglia [27]. Confocal microscopy analysis of trigeminal ganglion slices immunostained for Iba1 revealed that macrophages of KI ganglia in situ displayed, on average, a significantly (p<0.001) larger cell volume (165 ± 7%; n= 70 cells) vs WT (n=67 cells), since they acquired an amoeboid morphology with limited number of processes, suggesting a change in their activation state, as found in vitro.

### Co-culturing trigeminal ganglia with exogenous macrophages

It seemed interesting to find out whether the neuroinflammatory profile of R192Q KI cultures could be increased by adding activated macrophages because one theory proposes that, during an acute migraine attack, there is a sterile inflammation of meninges with strong appearance of inflammatory cells like macrophages [12,13].

To investigate this issue with an *in vitro* model, peritoneal macrophages (MФ) harvested from mice previously injected with Brewer thioglycollate medium were co-cultured with trigeminal ganglia. While the house keeping gene GAPDH or actin was clearly expressed in peritoneal macrophage samples, RT- PCR data for Ca_V_2.1 showed no detectable signal in WT or KI mouse peritoneal macrophages (n=5 samples). Adding equivalent numbers (12 × 10^4^) of purified MФ (i.e. Iba1-positive cells were 91 ± 5% with respect to the DAPI-positive cells) to WT or KI cultures led, 48 h later, to a larger global number of Iba1-positive cells in KI cultures (Figure 1A right, B right), without change in the mRNA or protein expression of P2X3 receptor subunits (Figure 1D-F).

**Figure 2:**
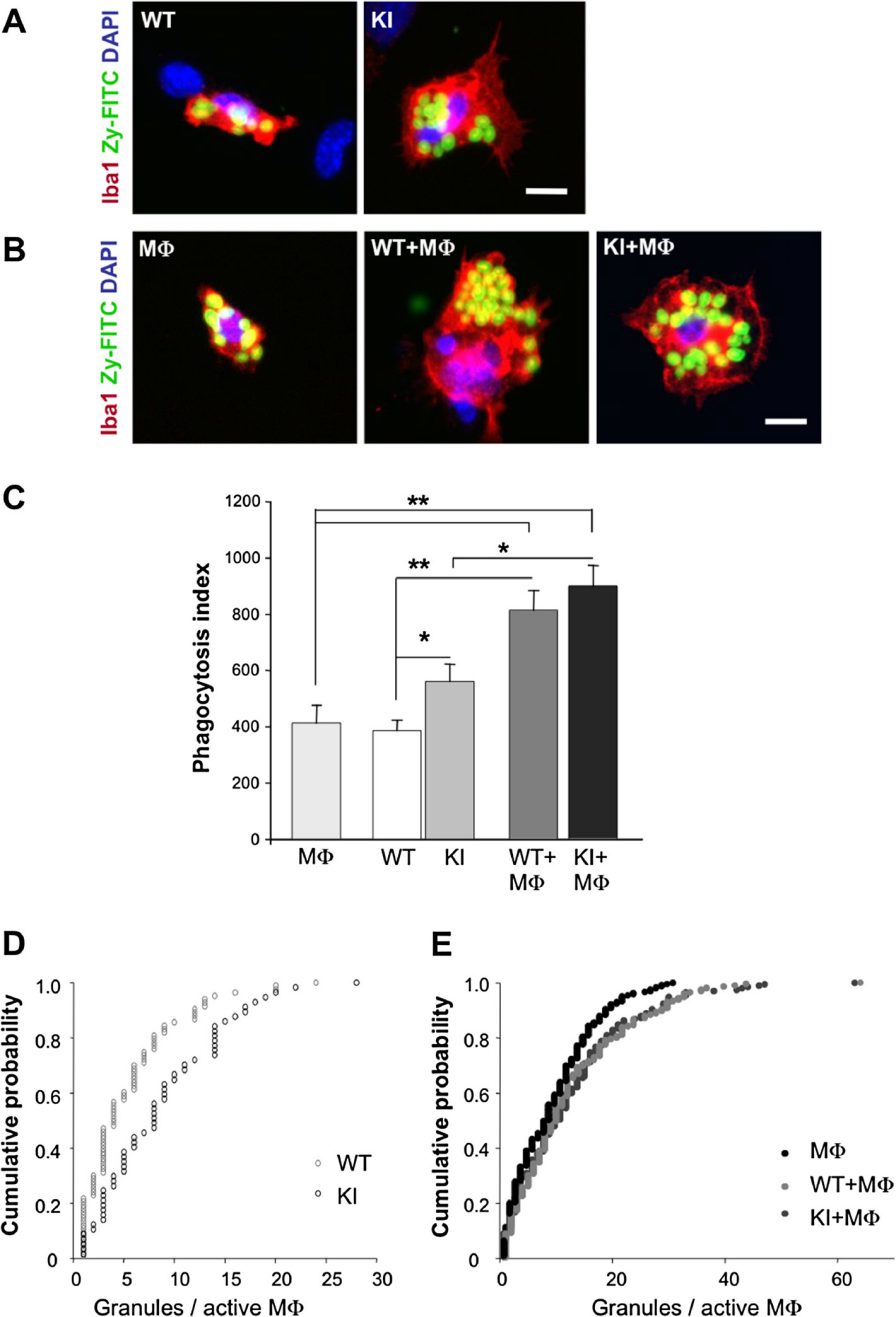
**Macrophage functional activity in culture. *A***, Examples of fluorescence microscopy images of macrophages (immunostained with anti-Iba1 antibody; red) in WT or R192Q KI trigeminal ganglion cultures. Active Iba1 immunoreactive macrophages show inclusion of round-shaped Zy-FITC granules (green). Cell nuclei are shown in blue (DAPI). Scale bar = 10 μm. ***B***, Images show peritoneal macrophages in culture (MФ). and macrophages/trigeminal co-cultures (WT+MФ and KI+MФ) processed like in ***A***. Scale bar = 10 μm. ***C***, Phagocytosis index for WT or R192Q KI cultures; *n* = 5 petri dish per group: * *p* < 0.05; ** *p* < 0.01. ***D, E***, Cumulative probability plots to calculate the occurrence of granule uptake in endogenous Iba1 cells in WT (grey triangles, *n* = 93 cells) or R192Q KI cultures (squares, *n* = 64) as well as for peritoneal macrophages (MФ, black circles, *n* = 241), and from co-cultures (WT+MФ. inverted triangles, *n* = 202. and R192Q KI (KI+MФ .diamonds, *n* = 234).

The basal activation state of macrophages was next evaluated in functional terms by using the phagocytosis test [22] calculated for the uptake of Zy-FITC granules added to the culture medium for 10 min. Figure 2A shows an example of images in which there was higher basal macrophage activation in R192Q KI than WT culture, consistent with the larger area of this cell type (Figure 1C). On average, R192Q KI macrophages had more Zy-FITC granules/active macrophage (9 ± 1) than WT macrophages (5 ± 1) (*n* = 5 petri dish per group, * *p* < 0.05; ** *p* < 0.01, Figure 2C). In accordance with Färber et al. [22], macrophage activity was expressed as phagocytosis index that was, on average, 390 ± 40 and 560 ± 60 for WT and R192Q KI cultures, respectively (Figure 2C). Cumulative probability plots to find out the occurrence of granule uptake in WT and R192Q KI cells are shown in Figure 2D in which the higher probability of detecting a larger number of granules was clearly assigned to R192Q KI cells, regardless of the actual number of granules. These data, therefore, provided a functional validation of higher constitutive macrophage activity in R192Q KI ganglion culture. Basal phagocytotic activity was detected in 75 ± 5% host MФ with an average value of 7.0 ± 1.5 granules/active macrophage (Figure 2B,C). These data were further validated by performing an uptake assay using inert fluorescent latex beads (Additional file 1: Figure S1) that provided very similar results. 

We explored whether co-culturing MФ with trigeminal ganglia might change macrophage phagocytosis activity at single cell level. Thus, Figure 2B shows that the number of granules taken up by single macrophages was larger in WT-macrophages co-cultures vs standard WT cultures. The same observation was obtained when comparing R192Q KI -macrophages co-cultures with R192Q KI cultures (Figure 2B). Thus, these observations enabled us to calculate the phagocytosis index that, for WT co-cultures, rose to 810 ± 70, a result similar to the one for KI co-cultures (900 ± 70; Figure 2C), suggesting that in both conditions a similar, strong functional activity was reached by adding macrophages. The next issue to be studied was whether macrophages could be divided into different cell clusters in terms of their phagocytotic activity, or whether there was a continuum of granule uptake process. This question was addressed as shown in Figure 2E that depicts cumulative plots for granule uptake process by host macrophages alone, or co-cultured with WT or R192Q KI cells, demonstrating that, in either type of co-culture, there was equal probability for analogous granule uptake. Thus, the different probability shown in Figure 2D was lost following co-culturing conditions (Figure 2E), indicating that, when tested under co-culturing conditions, the granule uptake by single macrophages was always enhanced.

### P2X3 receptor-mediated responses in the presence of host macrophages

**Figure 3:**
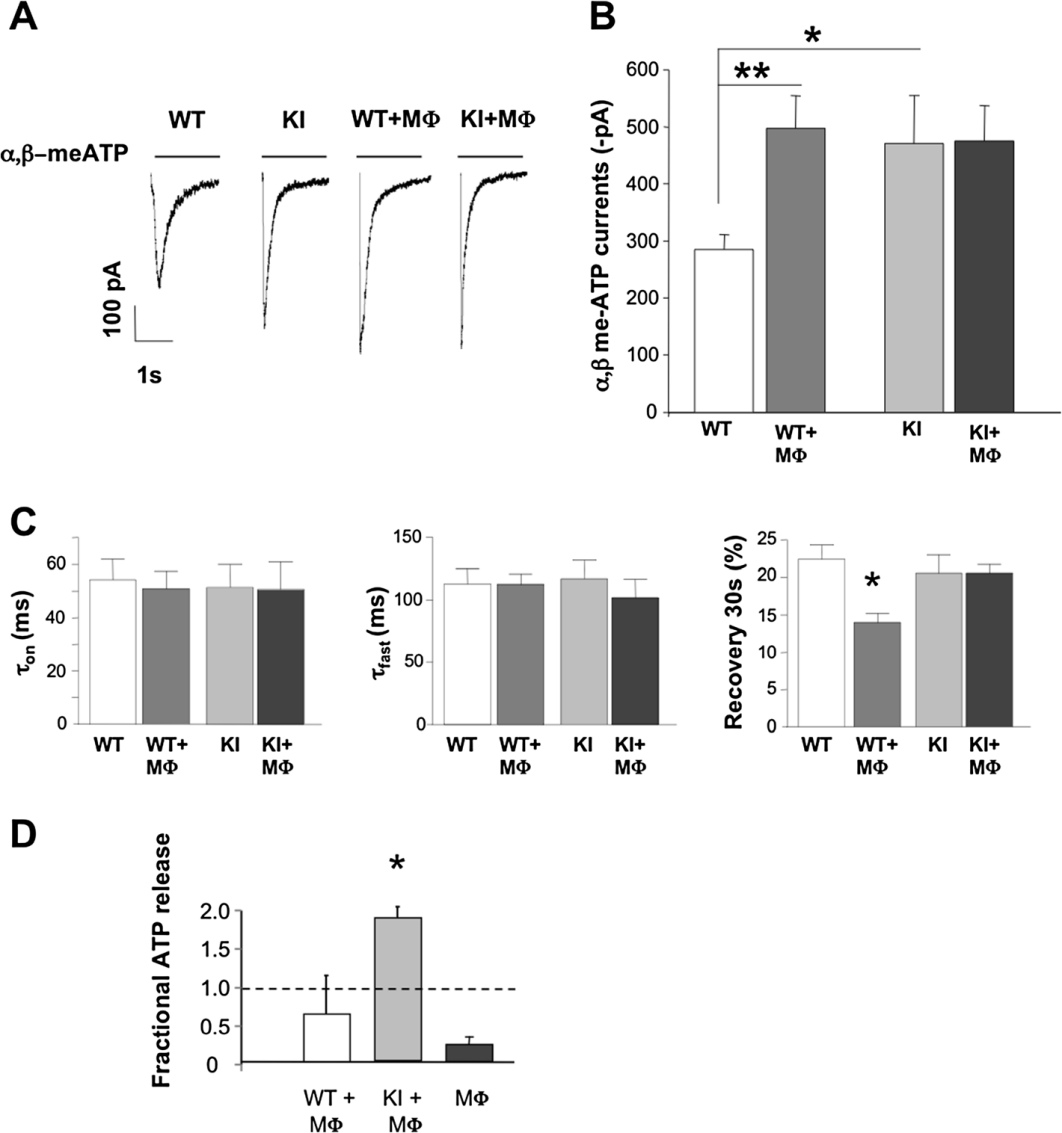
**Neuronal P2X3 receptor-mediated responses in basal conditions or in the presence of host macrophages. *A***, Representative examples of currents induced by application of α,β-meATP (10 μM, 2 s; horizontal bar) to trigeminal neurons from WT (*n* = 15 neurons) or R192Q KI (*n* = 17) cultures in standard conditions (left traces) or when co-cultured with macrophages (WT+MФ, .*n* = 24; KI+MФ, .*n* = 20. Note that macrophage co-culturing increases P2X3-mediated responses from WT neurons. Average data are plotted in ***B***. * *p* < 0.05; ** *p* < 0.01. ***C,*** Rise time (left; expressed as time from 10 to 90% of peak amplitude), desensitization onset (middle; expressed as the first time constant, τ_fast_, of current decay). Values are from 13-23 neurons. Recovery from desensitization (right; expressed as % of control amplitude in a paired pulse agonist application) was faster for WT+MФ vs WT; * *p* = 0.007. All responses were evoked by α,β-meATP (10 μM, 2 s). ***D***, ATP medium content measured 5 h in WT or KI trigeminal neuron-macrophage co-cultures. Basal ATP levels present in culture macrophages are also shown. Data are expressed as fractional increase with respect to neuronal WT or KI cultures. *n* = 2, * *p* < 0.05.

Figure 3A shows examples of currents induced by 2-s application of the selective P2X3 receptor agonist α,β-meATP (10 μM) to WT or R192Q KI neurons when cultured in standard conditions or co-cultured with MФ. As previously reported [8], R192Q KI neuronal currents were larger than those recorded from WT neurons (Figure 3B). When WT ganglia were co-cultured with host macrophages, a significant potentiation of P2X3 mediated currents was observed in WT neurons that expressed peak current values analogous to those normally seen in KI neurons. However, no further enhancement was detected in R192 KI neurons by adding host macrophages (Figure 3A,B). Other parameters of P2X3 receptor function, such as current rise-time (τ_on_; Figure 3C, left) and desensitization onset (τ_fast_; Figure 3C, middle) were not significantly changed by MФ co-culturing (*p* > 0.05). However, there was significant acceleration (tested at 30 s interpulse interval) of recovery from desensitization for WT + MФ vs WT alone (Figure 3C, right; WT 22 ± 2%, WT + MФ 14 ± 1%). A similar phenomenon did not occur for KI + MФ co-cultures (Figure 3C, right). 

A range of extracellular soluble factors, including ATP itself, might have been implicated in the crosstalk between sensory neurons and macrophages [28]. Hence, we measured basal ATP concentrations in WT or KI trigeminal cultures, and detected a higher level of ATP in the KI culture medium vs. the WT one (0.52 ± 0.06 pM for WT vs 1.26 ± 0.19 pM for KI cultures, n= 10 and 11 respectively, p= 0.0009). Furthermore, we investigated the impact (at 5 h; see also [29]) of adding macrophages on ATP release by KI and WT trigeminal cultures. Figure 3D shows that, while WT neuronal-macrophage co-cultures did not show significantly different ATP levels from control (*p* > 0.05), KI + MФ co-cultures released significantly more ATP (*p* < 0.05).

## Discussion

The principal finding of our study is that macrophages and sensory ganglion neurons could crosstalk with mutual facilitation of their functional activity. Since this process was observed in culture, it suggests that such an interaction may occur even without the intervention of blood-borne substances or cells.

### Basal macrophage profile of ganglion cultures

Notwithstanding the limitations of using a simple culture model, the present reports attempts to fill a gap between two distinct sets of data concerning the basic pathophysiology of migraine pain. On the one hand, the proposal of sterile inflammation predicts, at least in coincidence with the headache attack, the activation of inflammatory cells, among which macrophages are likely to play a major role [30], as contributors to sensitize nociceptive neurons via local release of substances [12,13]. This theory, however, does not clarify if a neuroinflammatory profile lingers in the absence of attack, if it may predispose to it, and what soluble mediators might trigger it.

On the other hand, the R192Q KI mouse model expressing a dysfunctional channelopathy affecting a certain class of neuronal Ca^2+^ channels, suggests a neuronal origin of migraine pain [31], but it does not fully identifies the mechanisms responsible for the actual pain onset or whether a causative role for non-neuronal cells is mandatory.

The present data suggest that R192Q KI ganglia and cultures constitutively contained a significantly larger number of macrophages with typical morphology of activation in comparison with WT cultures. Despite the fact that Iba-1 negative satellite glial cells have a key role in neuronal-glial crosstalk in trigeminal ganglia [11], the Iba1 positive cells investigated in the present report appeared to be a potential, new contributor to sensory neuron modulation even if they were a small minority in the global ganglion cell population. This notion was supported by the stronger phagocytosis index of such cultures. We propose that R192Q KI trigeminal ganglion cultures basally possessed an intrinsic milieu that predisposed macrophage activation, even if future work is necessary to identify the molecular mechanisms. A parsimonious hypothesis would be that the enhanced excitability of R192Q KI neurons detected even *in vitro*[8] was instrumental in determining the macrophage activation perhaps via release of soluble factors. To further test the potential crosstalk between neurons and macrophages, we performed experiments by adding exogenous macrophages to cultures, and observing the functional outcome.

### Co-culturing peritoneal macrophages and trigeminal sensory ganglia stimulated phagocytosis

Purified peritoneal macrophages had, on average, the same phagocytic activity of macrophages resident in WT trigeminal ganglion cultures. Co-culturing peritoneal macrophages with trigeminal ganglion cultures significantly enhanced the macrophage activity at single cell level, suggesting that either through macrophage mutual interaction or neuronal influence, single macrophages had acquired stronger function. This phenomenon was similarly detected with WT or R192Q KI co-cultures, suggesting that a specific neuronal phenotype was not necessary to potentiate macrophage activity at single cell level. Importantly, peritoneal macrophages did not express Ca_V_2.1 channels, thus making unlikely the possibility of a different contribution by WT or KI macrophages *per se*, once added to cultures. It is noteworthy that, even after co-culturing, the relative percentage of macrophages with respect to the total cell population was only 10–15%, suggesting that there was no large unbalance of cell types following co-culturing. Using the co-culturing protocol, granule uptake reached a plateau value within the time-course of our experimental observations.

### Influence of macrophages on neuronal responses mediated by P2X3 receptors

Under basal conditions, the higher number of macrophages of R192Q KI cultures had no effect on P2X3 receptor expression levels, even though P2X3 receptor responses were constitutively larger.

The co-culturing protocol significantly increased the responses of WT receptors that grew in amplitude to the constitutive level expressed by R192Q KI receptors. WT P2X3 receptors in co-culture, however, recovered less promptly from desensitization: the cause for this difference remains unclear, but it might be related to the action of yet unidentified soluble factors that may selectively affect certain phases of receptor desensitization [9,32]. It seemed feasible that, in the microenvironment generated by co-culturing macrophages with trigeminal ganglia, one contributor to the observed responses was ambient ATP [9,27,28,32] For this purpose, we measured extracellular ATP whose basal levels were rather small (pM range), yet significantly higher in KI cultures, a phenomenon even more evident in macrophages co-cultures. These data should, however, be interpreted with caution as endogenously-released compounds are strongly diluted in the large extracellular space of the cultures and are subjected to enzymatic breakdown and/or cell transport. Thus, the effective concentration of ATP at cell membrane level remains unknown. Notwithstanding these important limitations, it is plausible to assume that ambient ATP could modulate P2X3 receptor responsiveness via high affinity desensitization and trap a few receptors in a desensitized state with minimal influence on current peak [33]. This possibility would not readily explain macrophage-mediated enhancement of P2X3 receptor currents, in particular in WT neurons. A more likely explanation that, however, requires future investigation is that any effect by ambient ATP was indirectly generated via activation of highly sensitive P2Y receptors expressed by satellite glial cells that can shape P2X3 receptor function in trigeminal culture [11,27]. Furthermore, it is well established that extracellular ATP is not the only molecule that can modify P2X3 receptor kinetic properties and trigeminal neuronal responses [10,34]. Thus, it is hypothesized that ATP-evoked release of other soluble mediators (or active byproducts of ATP breakdown like ADP and adenosine) might have contributed to the observed effects.

The strong P2X3 responses generated by R192Q KI trigeminal ganglion neurons were not further augmented by co-culturing, indicating likely saturation of this process. In support of this notion, adding larger numbers of macrophages did not increment WT P2X3 receptor currents beyond the level observed in R192Q KI neurons. Likewise, there was no change in receptor desensitization properties which, in R192Q KI neurons, are regulated by a complex balance between intracellular kinases and phosphatases controlled by soluble extracellular factors [9].

We surmise that, for R192Q KI neurons, constitutive macrophages could contribute to potentiated responses mediated by P2X3 receptors, and that no further enhancement was seen merely by raising macrophage numbers.

## Conclusions

Assuming that the present data might be applicable to the *in vivo* condition, one can hypothesize that the larger macrophage population might create a local milieu that predisposes to the onset of P2X3 receptor mediated pain. Hence, macrophages and, probably, other inflammatory cells might together with satellite glial cells [11,35] concur to shift trigeminal neuronal sensitivity to a hyperfunctional state in a genetic model of migraine.

## Abbreviations

α,β-meATP: α,β-methyleneadenosine 5^′^-triphosphate; ATP: Adenosine-5^′^-triphosphate; AU: Arbitrary unit; BCA: Bicinchoninic Acid; β-tub III: β Tubulin III; Ca_V_2.1: Voltage activated calcium channel 2.1; CTR: Control; FHM-1: Familial hemiplegic migraine type 1; FITC: Fluorescein isothiocyanate; Iba1: Ionized calcium binding adaptor molecule 1; MФ: Peritoneal macrophage; KI: Knock-in; P2X3: Purinergic ionotropic receptor 3; ROI: Region of interest; RT-PCR: Real time polymerase chain reaction; WT: Wild-type; Zy-FITC: Zymosan FITC-conjugated.

## Competing interests

The authors declare that they have no competing interests.

## Authors’ contributions

AF molecular biology data; AN functional studies; TB ATP assays, AMJMVDM design and supply of genetic model; AN and EF, project supervision; AF, AMJMVDM, EF and AN joint contribution to MS writing. All authors read and approved the final manuscript.

## Supplementary Material

Additional file 1 **Figure S1. **Macrophage microspheres phagocytosis activity. Histograms quantifies the fluorescence values (arbitrary units; AU) of microspheres latex FITC-conjugated beads phagocytosis in peritoneal macrophage cultures (MФ., or trigeminal WT or KI cultures alone or in macrophage-neuronal co-cultures (WT+MФ and KI+MФ) *n* = 3, * *p* < 0.05; ** *p* < 0.01. Click here for file
