# Supplementary figures and images for: Functional crosstalk in culture between macrophages and trigeminal sensory neurons of a mouse genetic model of migraine
Source: BMC Neurosci. 2012 Nov 21;13:143. doi: 10.1186/1471-2202-13-143 (PMC3511260; doi:10.1186/1471-2202-13-143)

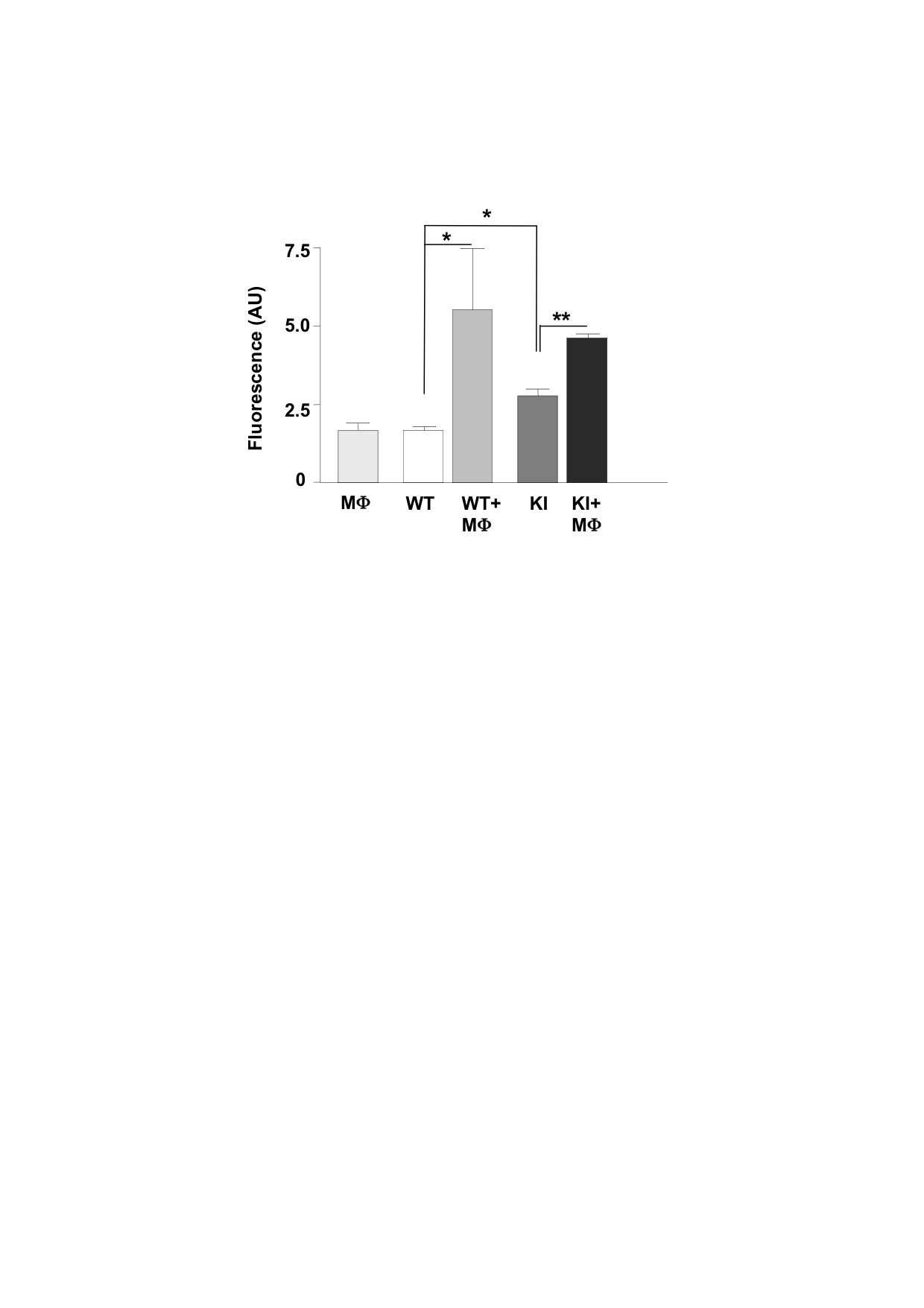

Supplement: Additional file 1 — Figure S1. Macrophage microspheres phagocytosis activity. Histograms quantifies the fluorescence values (arbitrary units; AU) of microspheres latex FITC-conjugated beads phagocytosis in peritoneal macrophage cultures (MФ., or trigeminal WT or KI cultures alone or in macrophage-neuronal co-cultures (WT+MФ and KI+MФ) n = 3, * p < 0.05; ** p < 0.01. [file 1471-2202-13-143-S1.tiff]
